# Warfarin Use Is Associated with Increased Mortality at One Year in Patients with Idiopathic Pulmonary Fibrosis

**DOI:** 10.1155/2021/3432362

**Published:** 2021-11-25

**Authors:** Syeda Fatima Naqvi, Amir Humza Sohail, Dhairya A. Lakhani, James Maurer, Sarah Sofka, Yousaf B. Hadi

**Affiliations:** ^1^Section of Pulmonary and Critical Care Medicine, Department of Medicine, West Virginia University, USA; ^2^Department of Surgery, NYU Langone Health, NYU Long Island School of Medicine, New York, USA; ^3^Department of Radiology, West Virginia University, USA; ^4^Section of Internal Medicine, Department of Medicine, West Virginia University, USA; ^5^Section of Gastroenterology and Hepatology, Department of Medicine, West Virginia University, USA

## Abstract

**Objectives:**

We studied the safety and efficacy of warfarin compared to direct acting oral anticoagulant use in patients with IPF.

**Methods:**

We conducted a retrospective cohort study of all patients with IPF who were prescribed warfarin or direct acting oral anticoagulants (DOACs) for cardiac or thromboembolic indications and followed at our institute for their care. Univariate tests and multivariable logistic regression analyses were used for assessing association of variables with outcomes.

**Results:**

A total of 73 patients were included in the study with 28 and 45 patients in the warfarin and DOAC groups, respectively. Univariable analysis revealed a significant difference in mortality in one year between warfarin and DOAC groups (7/28 vs. 3/45, *p* value 0.027). Significantly more patients in the warfarin group suffered an exacerbation that required hospitalization within one year (9/28 vs. 5/45, *p* value 0.026). Multivariate logistic regression analysis showed that anticoagulation with warfarin was independently associated with mortality at one-year follow-up (OR: 77.4, 95% CI: 5.94–409.3, *p* value: 0.007).

**Conclusion:**

In our study of patients with IPF requiring anticoagulants, we noted statistically significant higher mortality with warfarin anticoagulation when compared to DOAC use. Further larger prospective studies are needed to confirm these findings.

## 1. Introduction

Idiopathic pulmonary fibrosis (IPF) is a pulmonary disease of unknown etiology that is characterized by interstitial fibrotic change in lung parenchyma [1–3]. IPF carries a high morbidity and mortality, and no pharmacological agent has yet been shown to improve survival. Prior studies have demonstrated a role of the coagulation cascade in the pathogenesis of this disease [4, 5]. Based on this premise, its role as a therapeutic target was explored by a placebo-controlled trial of warfarin therapy for the treatment of IPF [6]. However, this trial was discontinued early due to excess mortality in the warfarin group. The increased mortality observed in this trial with warfarin use was not attributed to increase bleeding risk secondary to anticoagulation. The ACE-IPF trial mentioned above studied the role of warfarin for treatment of IPF in patients with no indications for anticoagulation. Therefore, major societies recommend against using warfarin for the treatment of IPF itself [7, 8]. However, the guideline recommends that patients with IPF who have an indication for anticoagulation should still be anticoagulated. IPF is associated with an increase in thromboembolic and acute coronary syndromes [9–11], with a recent meta-analysis showing a twofold increase in the risk of VTE in this patient population [12]. Warfarin continues to be used for thromboembolic and cardiac indications in these patients. No data exists in the current published scientific literature studying the comparative safety of warfarin with other anticoagulants in patients with IPF. Furthermore, there have been no prior studies exploring direct acting oral anticoagulants (DOACs) as a treatment modality for IPF, and data regarding safety of DOACs in this patient population, even for patients with indications for anticoagulation, is scarce. Therefore, the above-mentioned guideline recommendation does not mention use of DOACs. We performed a retrospective cohort study of patients with IPF with an indication for anticoagulation at our institution who were initiated on anticoagulation to compare the safety of warfarin with direct acting oral anticoagulants (DOACs).

## 2. Materials and Methods

### 2.1. Study Design and Population

We conducted a retrospective cohort study of all patients with a diagnosis of IPF at West Virginia University Hospital, a quaternary care referral center, who were initiated on anticoagulation for any indication during the period 2009-2019. The study was reviewed and approved by our institutional review board before commencement. Patients with IPF who were initiated on anticoagulation with warfarin or DOACs including apixaban, rivaroxaban, or dabigatran were identified using our electronic health record system, and their charts were reviewed by two study authors. Patients were included if they were following at our institution for IPF and were initiated on anticoagulation at our institute. Patients with no follow-up and those without confirmed diagnosis of IPF were excluded. At our institution, patients are diagnosed with IPF based on American Thoracic Society Clinical Practice Guideline [13].

### 2.2. Study Data

Variables of interest were extracted from patient charts by two study authors, including demographic characteristics (age, gender, ethnicity), smoking status, comorbid conditions, baseline pulmonary function tests, indication for anticoagulation, and type of anticoagulant used and disease exacerbations, major bleeding, and mortality in one year after initiation of anticoagulation, antifibrotic agent, and antiplatelet agent use, among others.

### 2.3. Study Outcomes

Primary endpoint of the study was mortality during one year of follow-up after being initiated on anticoagulation. Secondary outcomes included any disease exacerbations and major bleeding events (defined as bleeding requiring hospitalization, bleeding requiring transfusion, intracranial bleeding, and gastrointestinal bleeding) during follow-up. Clinic and hospital charts were reviewed. Patients were judged to have disease exacerbation for the purposes of our study if pulmonologist note from clinic or hospital judged them as suffering from IPF exacerbation.

### 2.4. Statistical Analyses

Patients were divided into two groups based on type of anticoagulant used (warfarin vs. DOAC). Demographic variables, clinical variables, and study outcomes were compared between the two groups. Bivariate analyses were conducted using *t*-tests, Fisher exact tests, and Chi square tests for continuous and categorical variables. A multivariable logistic regression model was fitted to assess independent associations of variables with the primary outcome of mortality at one-year follow-up and to control for confounders. A separate backward stepwise logistic regression model was applied to eliminate any bias from overfitting. The R statistical software was used to conduct all analyses [14], and *p* values less than 0.05 were considered significant for the purposes of this study. Subsequently, Kaplan Maier survival curves were plotted for the warfarin and DOAC groups, and survival in the two groups was compared using Log Rank Test. Patients were censored at mortality, discontinuation of anticoagulation, or completion/loss of follow-up.

## 3. Results

301 charts of patients with IPF were reviewed for inclusion. A total of 73 patients with IPF who were initiated on anticoagulation during the study period with complete records available were included (5 patients with incomplete records/no follow-up were excluded). The sample comprised a majority of male patients (54.8%), and 13 patients had a history of smoking (17.8%). Of the sample population, 28 patients (38.4%) were initiated on warfarin, while 45 patients (61.6%) were prescribed DOACs. In the DOAC group, apixaban was the most prescribed anticoagulant (30 patients) followed by rivaroxaban (15 patients). No patient received dabigatran or edoxaban.

The mean diffusion capacity of the lung (DLCO) of the sample at the time of initiation of anticoagulation was 43.29 (SD: 13.04) (40.33 ± 16.11 and 45.16 ± 10.74 in the warfarin and DOAC group, respectively). Antifibrotic agents were being used by 30 patients (41.1%). Approximately one-third of the patients (32.9%) were on home oxygen at the time of initiation of anticoagulation. Baseline characteristics of the sample are summarized in Table 1 and are compared between the two anticoagulant groups. More patients in the DOAC group were already requiring home oxygen at the time of initiation of anticoagulation (*p* = 0.03), while more patients in the warfarin group were on concurrent antiplatelet therapy (*p* = 0.01).

A total of 17 patients (23.3%) died during follow-up. Among these, 10 patients (13.7%) died within 1 year after initiation of anticoagulation. Major bleeding events were noted in 6 patients during follow-up while on anticoagulation (2 in the warfarin and 4 and DOAC group). Two patients in the warfarin and one patient in the DOAC group suffered major bleeding events within one year of anticoagulation initiation. There was no difference in rates of bleeding in the two groups, as detailed in Table 2. Major bleeding events were observed in four patients taking antifibrotic agents. Bleeding events were noted in 2 patients each taking Nintedanib and Pirfenidone during follow-up (no statistical difference, *p*: 0.66). In the total cohort, two patients suffered lower gastrointestinal bleeding. One patient experienced upper gastrointestinal bleeding. One patient each suffered retroperitoneal hematoma, hematuria, and pulmonary hemorrhage.

Univariable analyses revealed a significant difference in mortality in one year between warfarin and DOAC groups (7/28 vs. 3/45, Chi square *p* value 0.027) (Table 3). There was no difference between number of patients experiencing any exacerbation at one year between the two groups (9/28 vs. 7/45, *p* value 0.096); however, significantly more patients in the warfarin group suffered an exacerbation that required hospitalization within one year (9/28 vs. 5/45, Chi square *p* value 0.026). Univariable analyses comparing outcomes in the warfarin and DOAC groups are detailed in Table 2.

Multivariable logistic regression analysis was conducted for mortality in one year, incorporating age, gender, smoking history, antifibrotic agent use, history of malignancy, home oxygen use at initiation, presence of chronic obstructive pulmonary disease (COPD), concurrent antiplatelet use, and type of anticoagulant as covariates. Anticoagulation with warfarin was found to be independently associated with mortality at one-year follow-up (OR: 77.4, 95% CI: 5.94–409.3, *p* value: 0.007). History of malignancy was also associated with mortality at one year (OR: 36.45, 95% CI: 3.61–453.21, *p* value: 0.02). Associations of different variables with mortality at one year on multivariable and univariable analyses are given in Table 3.

To remove any bias from overfitting, a separate backward stepwise logistic regression was applied that included all variables given in Table 1 as covariates. The model successively eliminated variables from each step of the regression analysis, progressively removing variables with *p* values greater than 0.1. Warfarin use and history of malignancy were the only significant variables identified by the model (*p* values 0.005 and 0.009, respectively). Gender was also included in the final step of the model but failed to reach statistical significance (male gender, *p* = 0.062).

**Figure 1:**
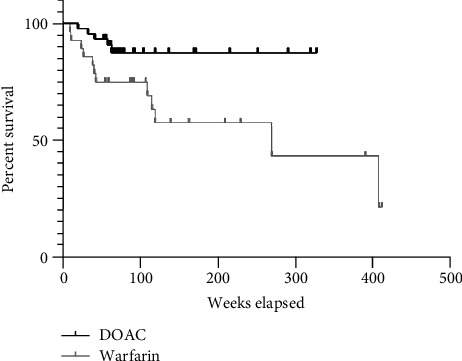
Survival proportions in the two study groups.

Figure 1 is a Kaplan Meier survival plot comparing the survival trend of the two groups during the follow-up period. Log Rank Test Hazard Ratio for warfarin was calculated to be 3.052 (95% confidence interval: 1.17–7.95, Log-rank Mantel Cox *p* value 0.02).

Anticoagulation failure was encountered in one patient each in the warfarin and DOAC group. Both patients had suffered an embolic stroke while being anticoagulated for atrial fibrillation.

3 patients had discontinuation of anticoagulation due to bleeding within first year, and a further 5 patients discontinued anticoagulation after completion of therapy at 6 months (these patients were censored after completion of therapy in the Kaplan Maier/Log Rank analysis). 10 patients died within 1 year as discussed above. The rest of the included cohort of patients were initiated on indefinite anticoagulation and received anticoagulation for more than 1 year.

## 4. Discussion

Large epidemiological studies have found an increase in thromboembolic phenomenon and acute coronary syndromes in patients with IPF, and therefore, these patients are frequently prescribed anticoagulant agents [8–11]. We found that a significant increase in one-year mortality in patients with IPF who were initiated on warfarin when compared to those taking DOACs. This finding adds to the existing data, most importantly from the ACE-IPF trial, regarding the safety of warfarin in IPF patients. The ACE-IPF trial randomized patients with IPF to receive either warfarin or placebo and found an increase in mortality in the warfarin arm [6]. In a disease with a very high mortality and unpredictable disease course, all interventions that may have a potential to worsen outcomes need to be very carefully weighed, and alternatives should be considered. Our study adds crucial evidence against the choice of warfarin as an anticoagulant for patients with IPF requiring anticoagulation.

The increase in mortality seen with warfarin use in our cohort seems to be independent of bleeding events and bleeding associated complications; in fact, the bleeding rate in the two groups was similar. This is consistent with the ACE-IPF trial which also had no bleeding events in either group [6]. Additionally, of the 14 deaths reported in the warfarin arm in the ACE-IPF trial, 11 were secondary to respiratory events.

Protein C has been postulated to play a role in this worsening mortality seen with warfarin use in IPF. Levels of protein C have been found to be low in patients with IPF, creating a procoagulant state in the lung parenchyma [15]. Administration of intratracheal protein C in mice treated with bleomycin reduced lung fibrosis. Mice treated with protein C and bleomycin had less fibrotic lesions in the central and subpleural areas of the lung as opposed to those treated with bleomycin alone [16]. Protein C is a vitamin K dependent factor; warfarin inhibits vitamin K epoxide reductase thereby preventing activation of vitamin K dependent factors. Warfarin administration may further deplete levels of protein C in the lungs, removing this protective effect of protein C against fibrosis.

Matrix gla protein (MGP) is another vitamin K dependent protein, and low levels of MGP may also play a role in IPF exacerbations. In the extracellular matrix, MGP prevents calcification [17] and is dependent on vitamin K for activation. Warfarin inhibits vitamin K activation, leading to depletion of active MGP which leads to accelerated calcification [18]. As opposed to warfarin, DOACs and heparin products do not affect vitamin K activation, and therefore, activation of protein C and MGP is not affected by the use of these agents.

The hypothesis that a procoagulant lung environment in IPF promotes fibrosis, and disease progression, has led to exploration of the coagulation cascade as a therapeutic target. Of the two trials conducted in this realm, ACE-IPF, discussed above, was stopped early due to excess mortality in the warfarin arm. Patients randomized to warfarin experienced more hospitalizations and disease-related exacerbations compared to placebo. However, an unblinded small Japanese trial exploring anticoagulation in IPF found a decrease in mortality in the warfarin group. In that study, patients in the anticoagulant group received warfarin and prednisolone in the outpatient setting but were administered low-molecular weight heparin (LMWH) in the inpatient setting [19]. It has been postulated that their results may be explained by the use of LMWH in their study. Apart from its anticoagulant action, LMWH also inhibits lysyl oxidase, which prevents cross linkage of collagen and elastin in extracellular matrix [20]. Alagha and colleagues [21] described two cases of patients with IPF who experienced significant exacerbations of their disease after initiation of warfarin that ultimately resulted in death; the first patient experienced some improvement in symptoms after warfarin was discontinued and replaced by heparin with the initiation of steroids. Regardless of a role in disease pathogenesis and potential role in treatment of IPF itself, heparins have not been associated with adverse outcomes in patients with IPF who have an indication for anticoagulation.

King et al. studied the association between anticoagulation and survival in interstitial lung disease. Patients were recruited from the Pulmonary Fibrosis Foundation registry [22]. In their registry analysis which included 106 patients with idiopathic pulmonary fibrosis (IPF) initiated on anticoagulation (49 warfarin; 57 DOACs), higher mortality was found in the anticoagulation group. Direct acting oral anticoagulants (DOACs) and warfarin were associated with twofold and 2.5-fold risk of death/transplant compared with the nonanticoagulation group, respectively. Authors noted a trend towards worse survival with warfarin compared to DOACs, but this analysis failed to reach statistical significance. Due to registry-based nature of data, authors could not determine duration of anticoagulation. Prescription of anticoagulant was taken as index event with censoring performed at death or end of follow-up. Many patients would have discontinued anticoagulation during the follow-up period, and ideally, censoring of events for the cox-proportional hazard analysis should be performed at anticoagulant discontinuation (end of exposure). Inclusion of follow-up period in the model beyond the time of anticoagulant discontinuation thus would have diluted any possible effect of warfarin potentially leading to loss of significance in the DOAC vs. warfarin analysis. In our study, we were able to account for duration of anticoagulation and censor patients at time of discontinuation. Our study showed significant difference in mortality in one year between the groups (7/28 vs. 3/45, *p*: 0.027).

Previous data on safety of DOACs in this population was scarce. Our study now adds this data to the scientific pool as well. Our data suggests that heparins and DOACS are a safer alternative to warfarin in patients with IPF. Our study is limited by its retrospective study design and small sample size. The number of patients taking antiplatelet medications was significantly different between the two groups at baseline that can influence bleeding risk. However, the multivariable model in our study for the primary outcome incorporated antiplatelet use as covariate and should control for any confounding by concurrent antiplatelet medication use on mortality rate. Furthermore, there was no difference in bleeding rate between groups. Other limitations include lack of power analysis or sample size calculation before the study.

However, we were able to control for possible confounders in our patients, and a significant difference in mortality was found at one-year follow-up. Considering that alternate anticoagulants (DOACs) are now available and are now approved for most thromboembolic and cardiac indications, our data, along with data from the ACE-IPF, raise some concern regarding the safety of warfarin use in IPF. Further larger studies comparing warfarin use with DOACs in patients with IPF needing anticoagulation are needed to further explore the safety and efficacy of warfarin and DOACs in patients with IPF.

## Figures and Tables

**Table 1:** Baseline characteristics of the study population.

Variable	Total population	Warfarin group (*n* = 28)	DOAC group (*n* = 45)	*p* value
Age in years, mean (SD)	73.78 (11.25)	73.29 ± 11.67	74.09 ± 11.11	0.77
Male gender	40 (54.8%)	14 (50%)	26 (57.8%)	0.51
Comorbid conditions				
Diabetes	27 (37%)	14 (50%)	13 (28.9%)	0.07
Congestive heart failure	31 (42.5%)	13 (46.4%)	18 (40%)	0.59
Cerebrovascular disease	11 (15.1%)	5 (17.9%)	6 (13.3%)	0.60
Hypertension	49 (67.1%)	21 (75%)	28 (62.2%)	0.26
Coronary artery disease	38 (52.1%)	17 (60.7%)	21 (46.7%)	0.24
COPD	16 (21.9%)	5 (17.9%)	11 (24.4%)	0.51
History of malignancy	19 (26%)	5 (17.9%)	14 (31.1%)	0.21
Smoking history	13 (7.8%)	4 (14.3%)	9 (20%)	0.54
Antifibrotic agent use	30 (41.1%)	8 (28.6%)	22 (48.9%)	0.09
Nintedanib		3 (10.7%)	9 (20%)	
Pirfenidone		5 (17.8%)	13 (28.9%)	
Home oxygen use at initiation of anticoagulation	24 (32.9%)	5 (17.9%)	19 (42.2%)	0.03
DLCO at initiation of anticoagulation, mean (SD)	43.29 ± 13.04	40.33 ± 16.11	45.16 ± 10.74	0.37
Concurrent use of antiplatelet therapy	33 (45.2%)	18 (64.3%)	15 (33.3%)	0.01
Indication for anticoagulation				
Atrial fibrillation	45 (61.6%)	16 (57.1%)	29 (64.4%)	0.39
CHA₂DS₂-VASc (median, IQR)		4 (1.5)	4 (1.25)	
Thromboembolism	24 (32.9%)	8 (28.6%)	16 (35.6%)	0.79
Inherited coagulopathy with thrombotic event	4 (5.5%)	3 (10.7%)	1 (2.2%)	0.12

**Table 2:** Outcomes in the warfarin and DOAC groups.

Variable	Warfarin group (*n* = 28)	DOAC group (*n* = 45)	Univariable *p* value
All-cause mortality at 1 year	7 (25%) Respiratory failure (in hospital mortality) (3) Hospice care with subsequent demise (2) Pneumonia (1) Pulmonary hemorrhage (1)	3 (6.6%) Respiratory failure (in hospital mortality) 2 Hospice care with subsequent demise (1)	0.027
Any disease exacerbation at one year	9 (32.1%)	7 (15.5%)	0.096
Any disease exacerbation requiring hospitalization in 1 year	9 (32.1%)	5 (11.1%)	0.026
Major bleeding episode at one-year follow-up	2 (7.1%)	1 (2.2%)	0.303
Major bleeding event at any time during follow-up	2 (7.1%)	4 (8.89%)	0.79
Anticoagulation failure	1 (3.6%)	1 (2.2%)	0.73

**Table 3:** Association of baseline variables with mortality at one year, on bivariate and multivariate analyses (*p* values).

Variable	Bivariate analyses	Multivariable analyses
Age	0.12	0.13
Male gender	0.30	0.05
Smoking	0.85	0.41
COPD	0.88	0.61
Antifibrotic agent use	0.94	0.88
History of malignancy	0.008	0.024
Home oxygen use at initiation	0.61	0.81
Anticoagulation with warfarin	0.027	0.007
Antiplatelet agent use	0.72	0.16

## Data Availability

De-identified data can be made available upon reasonable request.

## References

[1] Fernández Pérez E. R., Daniels C. E., St Sauver J. (2010). Incidence, prevalence, and clinical course of idiopathic pulmonary fibrosis: a population-based study. *Chest*.

[2] King T. E., Tooze J. A., Schwarz M. I., Brown K. R., Cherniack R. M. (2001). Predicting survival in idiopathic pulmonary Fibrosis. *American Journal of Respiratory and Critical Care Medicine*.

[3] Navaratnam V., Fogarty A. W., McKeever T. (2014). Presence of a prothrombotic state in people with idiopathic pulmonary fibrosis: a population-based case–control study. *Thorax*.

[4] De Andrade J., Olman M. (2011). *Hemostasis and Fibrinolysis in the Pathogenesis of Lung Injury and Repair. Interstitial Lung Disease*.

[5] Günther A., Mosavi P., Ruppert C. (2000). Enhanced tissue factor pathway activity and fibrin turnover in the alveolar compartment of patients with interstitial lung disease. *Thrombosis and Haemostasis*.

[6] Noth I., Anstrom K. J., Calvert S. B. (2012). A placebo-controlled randomized trial of warfarin in idiopathic pulmonary fibrosis. *American Journal of Respiratory and Critical Care Medicine*.

[7] Raghu G., Rochwerg B., Zhang Y. (2015). An official ATS/ERS/JRS/ALAT clinical practice guideline: treatment of idiopathic pulmonary fibrosis. An update of the 2011 clinical practice guideline. *American Journal of Respiratory and Critical Care Medicine*.

[8] Cottin V., Crestani B., Valeyre D. (2013). French practical guidelines for the diagnosis and management of idiopathic pulmonary fibrosis. From the National Reference and the Competence centers for rare diseases and the Societe de Pneumologie de Langue Française. *Revue des Maladies Respiratoires*.

[9] Hubbard R. B., Smith C., le Jeune I., Gribbin J., Fogarty A. W. (2008). The association between idiopathic pulmonary fibrosis and vascular Disease. *American Journal of Respiratory and Critical Care Medicine*.

[10] Sprunger D. B., Olson A. L., Huie T. J. (2012). Pulmonary fibrosis is associated with an elevated risk of thromboembolic disease. *European Respiratory Journal*.

[11] Sode B. F., Dahl M., Nielsen S. F., Nordestgaard B. G. (2010). Venous thromboembolism and risk of idiopathic interstitial Pneumonia. *American Journal of Respiratory and Critical Care Medicine*.

[12] Boonpheng B., Ungprasert P. J. S. (2018). Risk of venous thromboembolism in patients with idiopathic pulmonary fibrosis: a systematic review and meta-analysis. *Sarcoidosis, Vasculitis, and Diffuse Lung Diseases*.

[13] Raghu G., Remy-Jardin M., Myers J. L. (2018). Diagnosis of idiopathic pulmonary fibrosis. An official ATS/ERS/JRS/ALAT clinical practice guideline. *American Journal of Respiratory and Critical Care Medicine*.

[14] RC Team (2013). *R: A Language and Environment for Statistical Computing*.

[15] Yasui H., Gabazza E. C., Taguchi O. (2000). Decreased protein C activation is associated with abnormal collagen turnover in the intraalveolar space of patients with interstitial lung disease. *Clinical and Applied Thrombosis/Hemostasis*.

[16] Yasui H., Gabazza E. C., Tamaki S. (2001). Intratracheal administration of activated protein C inhibits bleomycin-induced lung fibrosis in the mouse. *American Journal of Respiratory and Critical Care Medicine*.

[17] Schurgers L. J., Uitto J., Reutelingsperger C. P. (2013). Vitamin K-dependent carboxylation of matrix Gla-protein: a crucial switch to control ectopic mineralization. *Trends in Molecular Medicine*.

[18] Booth A. J., Hadley R., Cornett A. M. (2012). Acellular normal and fibrotic human lung matrices as a culture system forIn VitroInvestigation. *American Journal of Respiratory and Critical Care Medicine*.

[19] Kubo H., Nakayama K., Yanai M. (2005). Anticoagulant therapy for idiopathic pulmonary fibrosis. *Chest*.

[20] Gavriel P., Kagan H. M. (1988). Inhibition by heparin of the oxidation of lysine in collagen by lysyl oxidase. *Biochemistry*.

[21] Alagha K., Secq V., Pahus L. (2015). We should prohibit warfarin in idiopathic pulmonary fibrosis. *American Journal of Respiratory and Critical Care Medicine*.

[22] King C. S., Freiheit E., Brown A. W. (2021). Association Between Anticoagulation and Survival in Interstitial Lung Disease: An Analysis of the Pulmonary Fibrosis Foundation Patient Registry. *Chest*.

